# High Glucose Induces Down-Regulated GRIM-19 Expression to Activate STAT3 Signaling and Promote Cell Proliferation in Cell Culture

**DOI:** 10.1371/journal.pone.0153659

**Published:** 2016-04-21

**Authors:** Yong-Guang Li, Bei-Bei Han, Feng Li, Jian-Wu Yu, Zhi-Feng Dong, Geng-Ming Niu, Yan-Wei Qing, Jing-Bo Li, Meng Wei, Wei Zhu

**Affiliations:** 1 State Key Discipline Division, Heart Center, Shanghai Jiao Tong University Affiliated Sixth People’s Hospital, Shanghai, People’s Republic of China; 2 Division of Cardiology, Zhejiang Provincial People’s Hospital, Hangzhou, Zhejiang, People’s Republic of China; 3 Provincial Key Laboratory of Cardiovascular Research, Department of Cardiology, Zhejiang University School of Medicine 2nd Affiliated Hospital, Hangzhou, Zhejiang, People’s Republic of China; University of South Alabama, UNITED STATES

## Abstract

Recent studies indicated that Gene Associated with Retinoid-IFN-Induced Mortality 19 (GRIM-19), a newly discovered mitochondria-related protein, can regulate mitochondrial function and modulate cell viability possibly via interacting with STAT3 signal. In the present study we sought to test: 1) whether GRIM-19 is involved in high glucose (HG) induced altered cell metabolism in both cancer and cardiac cells, 2) whether GRIM-19/STAT3 signaling pathway plays a role in HG induced biological effects, especially whether AMPK activity could be involved. Our data showed that HG enhanced cell proliferation of both HeLa and H9C2 cells, which was closely associated with down-regulated GRIM-19 expression and increased phosphorylated STAT3 level. We showed that GRIM-19 knock-down alone in normal glucose cultured cells can also result in an increase in phosphorylated STAT3 level and enhanced proliferation capability, whereas GRIM-19 over-expression can abolished HG induced STAT3 activation and enhanced cell proliferation. Importantly, both down-regulated or over-expression of GRIM-19 increased lactate production in both HeLa and H9C2 cells. The activated STAT3 was responsible for increased cell proliferation as either AG-490, an inhibitor of JAK2, or siRNA targeting STAT3 can attenuate cell proliferation increased by HG. In addition, HG increased lactate acid levels in HeLa cells, which was also observed when GRIM-19 was genetically manipulated. However, HG did not affect the lactate levels in H9C2 cells. Of note, over-expression of GRIM-19 and silencing of STAT3 both increased lactate production in H9C2 cells. As expected, HG resulted in significant decreases in phosphorylated AMPKα levels in H9C2 cells, but not in HeLa cells. Interestingy, activation of AMPKα by metformin was associated with a reversal of the suppressed GRIM-19 expression in H9C2 cells, the fold of changes in GRIM-19 expression by metformin were much less in HeLa cells. Metformin did not affect the phosphorylated STAT3 lelvels, however, decreased its levels in H9C2, especially in the setting of HG culture. Not like HG alone which resulted in no changes in lactate acid in H9C2 cells, metformin can increase lactate acid levels in H9C2 cells. Increased lactate induced by metformin was also observed in HeLa cells.

## Introduction

Diabetes mellitus is a common disease that exerts tremendous impact on human health. It has been shown that patients with diabetes are also at a significantly higher risk of developing various types of cancer [1]. Data has shown that approximately 80%of patients with pancreatic cancer suffer from hyperglycemia or diabetes[2]. And high glucose (HG) has been considered as a subordinate cause, that can trigger direct and/or indirect mechanisms to promote cancer cell proliferation, migration and survival [3,4]. However, the underlying mechanisms for this relationship are still not fully understood. Due to its clinical significance, increasing efforts have been made, trying to elucidate the link of carcinogenesis to the status of patients having high fasting glucose level, or being obese or diabetic [5,6], this is particularly important because an appropriate blood glucose level control could significant affect the occurrence and prognosis of cancer.

On the other hand, mitochondria has been shown to play important roles in cancer cells, maintaining mitochondrial potential and oxidative equilibrium that are essential for apoptosis and cell viability[7]. In fact, mitochondria is becoming an important therapeutic target for anticancer drug, such as mitocans, which can eventually cause cell death via interrupting mitochondrial integrity[8]. Recently, studies have shown that GRIM-19, also named NDUFA13, acts as a cell death-regulatory protein that can be induced by the combination of interferon-beta and retinoic acid [9]. GRIM-19 is also identified as one mitochondrial complex I subunit, which not only plays an important role in oxidative phosphorylation (OXPHOS) for ATP generation[9], but also is involved in the process of glycolysis, a key metabolic process for cancer[10]. Thus, GRIM-19 has the ability to modulate cancer cell survival. Data has shown that a mono-allelic loss of GRIM-19 can promote carcinogenesis in mice [11] and the tumor-derived mutations in GRIM-19 in human can also promote tumor growth in mice [12]. Moreover, GRIM-19 exerts the pro-survival effects through its interactions with signal transducer and activator of transcription-3 (STAT3)[13] which is an important member of the STAT family protein. In response to cytokines and growth factors, such as IL-6 and epidermal growth factor, STAT3 is activated through its phosphorylation at tyrosine 705 and forms homo- or hetero-dimers that translocate to the cell nucleus, acting as a transcription activator to regulate many cellular processes such as cell growth and apoptosis [13]. Interestingly, data has also linked STAT3 to both normal [14] and altered insulin signaling in the setting of diabetes [15]. Our previous study has indicated that STAT3 signaling was involved in HG induced HepG2 cells proliferation [16]. In fact, it has been well demonstrated that HG can exert toxic effects on normal organ cells, such as cardiac cells [17,18] as well as cancer cells [19,20,21], for which activity of AMPK was shown to be closely involved. However, it has not been shown whether Grim-19 is involved in the HG/STAT3 signaling, especially whether the altered metabolism could link Grim-19 expression to AMPK activity.

Therefore, in the present study using HeLa and H9C2 cell line, we sought to investigate: 1) the relationship between the expression level of GRIM-19 and activity of STAT3 signaling in the setting of HG; 2) how AMPK activity interact with Grim-19 in HG cultured cells and 3) how metformin, an AMPK agonist, modulates the expression of Grim-19.

## Materials and Methods

### Cell lines and treatment conditions

HeLa cells and H9C2 cells were obtained from the American Type Culture Collection (ATCC, Manassas, VA, USA). The HeLa cells and H9C2 cells were maintained in DMEM medium containing normal level (5.5 mM) or high (25 mM) level of glucose supplemented with 10% FBS, L-glutamine and antibiotics (100 units/ml penicillin, and 100 μg/ml streptomycin) at 37°C in the presence of 5% CO2. And the effect of IL-6 and the role of GRIM-19 in HeLa cells and H9C2 cells cultured in HG DMEM were investigated by plasmid that has been inserted either scramble or siRNA targeting GRIM-19.

### Reagents

The MTT Cell Proliferation and Cytotoxicity Assay kit was purchased from Sangon Biotech (Shanghai, PR China), AG490 from Beyotime(Jiangsu, PR China) and IL-6 from eBioscience (eBioscience, CA, USA). Primary antibodies against β-actin and GAPDH were purchased from Santa Cruz (Shanghai, China), antibody targeting GRIM-19 from Abcam (ab3449, Shanghai, PR China), and antibody against p- STAT3 (#4113), total STAT3 (#12640), p- AMPKα (Thr172, #2531), total AMPKα (#2532, p- Akt (Ser473, #4058) and total AKT (#4685) were from Cell Signaling Technology (Danvers, MA, USA). And all the secondary antibodies were purchased from Biosynthesis Biotechnology (Beijing, PRChina); penicillin, streptomycin, DMEM medium, and fetal bovine serum (FBS) were obtained from GIBCO (Grand Island, NY, USA).

### MTT assays *in vitro*

The effects of either HG and/or GRIM-19 over-expression on HeLa and H9C2 cells viability were determined by the nicotinamide,3-(4,5-dimetrylthiazol-2-yl)-2,5-diphenyltetrazolium bromide (MTT) dye uptake method. Briefly, the cells were equally distributed in 96-well plates at a density of 1 x 10^4^ cells/well (counted by a hemocytometer). Cells were treated with different glucose level for 48 hours. Before incubation with MTT, DMEM (serum10%) medium was removed and the final concentration of MTT (Sigma, St. Louis, MO, USA) at 5 mg/ml in DMEM medium and incubation was for 6 h in the dark at 37°C. Then, the supernatant was removed from each well. The colored formazan crystal produced from MTT was dissolved in 150 μL DMSO and cell viability was determined spectrophotometrically at 490 nm (BioTek, Vermont, USA).

### Western blotting analysis

Cells were harvested and washed with cold phosphate-buffered saline (PBS) and lysed in RIPA buffer (150 mM NaCl, 50 mM Tris-HCl, pH 7.2, 1% deoxycholic acid, 1% Triton X-100, 0.1% sodium dodecyl sulfate (SDS), 0.25 mM EDTA) with the protease inhibitor cocktail (Roche, Basel, Switzerland).The lysate was separated by SDS-polyacrylamide gel electrophoresis (SDS-PAGE) and transferred to NC membrane. The membrane was blocked with PBS containing 5% non-fat milk containing 0.05% Tween 20 followed by incubation with primary antibodies. Appropriate secondary antibodies were then used. The bound antibodies were visualized by enhanced chemiluminescene (Thermo, MA, USA). The band for each protein was then quantified by densitometry using Image J software (version 1.41, NIH, USA) and normalized to the expression of ß-actin or GAPDH for protein loading.

### Lactate acid quantification

A commercially available kit was used (Cat# A019-2, JianCheng BioTech, Nanjing, PR China) to measured the lactate acid level in cell culture medium that was obtained at the end of each cell experiment. All the data were expressed as fold changes in comparison to controls.

### Reverse transcription PCR analysis

After cells were transfected with GRIM-19 or siRNA-GRIM-19 for 48h, the cells were pre-treated with different levels of glucose for another 48 h. Total RNA was then isolated from the cells, using TRIzol® Reagent (Life Technologies, Rockville, MD, USA) following the manufacturer’s protocol. Complementary DNA was synthesized from 1 μg of total RNA using a cDNA Synthesis Kit (Takara Biotechnology Co., Ltd., China) following the manufacturer’s instructions. Primer sequences (Genecore, Shanghai, China) specific for CyclinB1, CyclinD1, Bcl-2, VEGF, STAT3 and GRIM-19 are shown in Table 1. β-actin was used for the loading control. After cDNA synthesis, the PCR reaction consisted of 32 cycles of denaturation at 95°C for 30 s, annealing at 56°C for 30 s, extension at 72°C for 30 s, and a further 5 min at 72°C in the last cycle. PCR products were separated by electrophoresis on a 2% agarose gel and visualized by staining with ethidium bromide. The expression was quantified densitometrically using the Gel Image Ver. 3.74 System (Tianon, Shanghai, China).

**Table 1:** Primer sequences for each targeted gene.

Name:	Sequence(5'-3'):	Product(bp):
CyclinB1	F:GCAGCACCTGGCTAAGAATGT	147
	R:GCCTTGGCTAAATCTTGAACT	
CycllinD1	F:GCGAGGAACAGAAGTGCG	484
	R:AGGCGGTAGTAGGACAGGAA	
Bcl-2	F:AGGATTGTGGCCTTCTTTGA	155
	R:CCTACCCAGCCTCCGTTAT	
VEGF	F:ACGGACAGACAGACAGACACC	176
	R:CCCAGAAGTTGGACGAAAAGT	
β-actin	F:AGCCTCGCCTTTGCCGATCC	100
	R:ACATGCCGGAGCCGTTGTCG	
STAT3	F:AGTCAGTGACCAGGCAGAAGA	265
	R:ATTTGTTGACGGGTCTGAAGT	
GRIM-19	F:CGGGACCGGAAGTGTGGGATAC	435
	R:GCAGAGCATTTATTCCGTCCCAG	

F: forward; R: reverse.

### Transient RNA interference and transfections

Both GRIM-19 and STAT3 were knocked down using small siRNAs, with non-targeting siRNA (scramble RNA) used in parallel as a negative control respectively (GenePharma Co., Shanghai, PR China). Primary cultured cells were transfected with siRNA (targeting either GRIM-19 or STAT3) or scramble RNA using Lipofectamine 2,000 (Invitrogen, Carlsbad, CA) according to the manufacturer’s instructions. Forty-eight hours after transfection of mRNA silencing, HeLa or H9C2 cells were collected for protein expression analysis of either GRIM-19 or STAT3 to confirm the effects of siRNA.

### Plasmid, lentiviral construction and DNA infection

The over-expression of GRIM-19 lentivirus was constructed (from Shanghai Sunbio Medical Biotechnology) which contained GFP and Flag. Human GRIM-19 sequence was amplified by RT-PCR from HeLa cells. WT-GRIM-19 with complete open reading frame was cloned into NOT I and EcoR V sites of mammalian expression vector Pflag-CMVTM-4. The pFLAG tag was added at the N-terminal of the GRIM-19 sequences in all constructs. Infection of plasmids to cells was performed using Lipofectamine 2,000 (Invitrogen, Carlsbad, CA) according to the manufacturer’s instructions. Forty-eight hours after infection, HeLa and H9C2 cells were analyzed for protein expression of GRIM-19 expression levels.

### Statistical analysis

Dates are expressed as means ± SD for three or more independent experiments. Statistical significance was estimated by one- way ANOVA followed by Student–Newman–Keuls test for comparison of several groups. P < 0.05 was considered statistically significant.

## Results

### Enhanced HeLa cell proliferation induced by HG was associated with down-regulated GRIM-19 level

**Fig 1:**
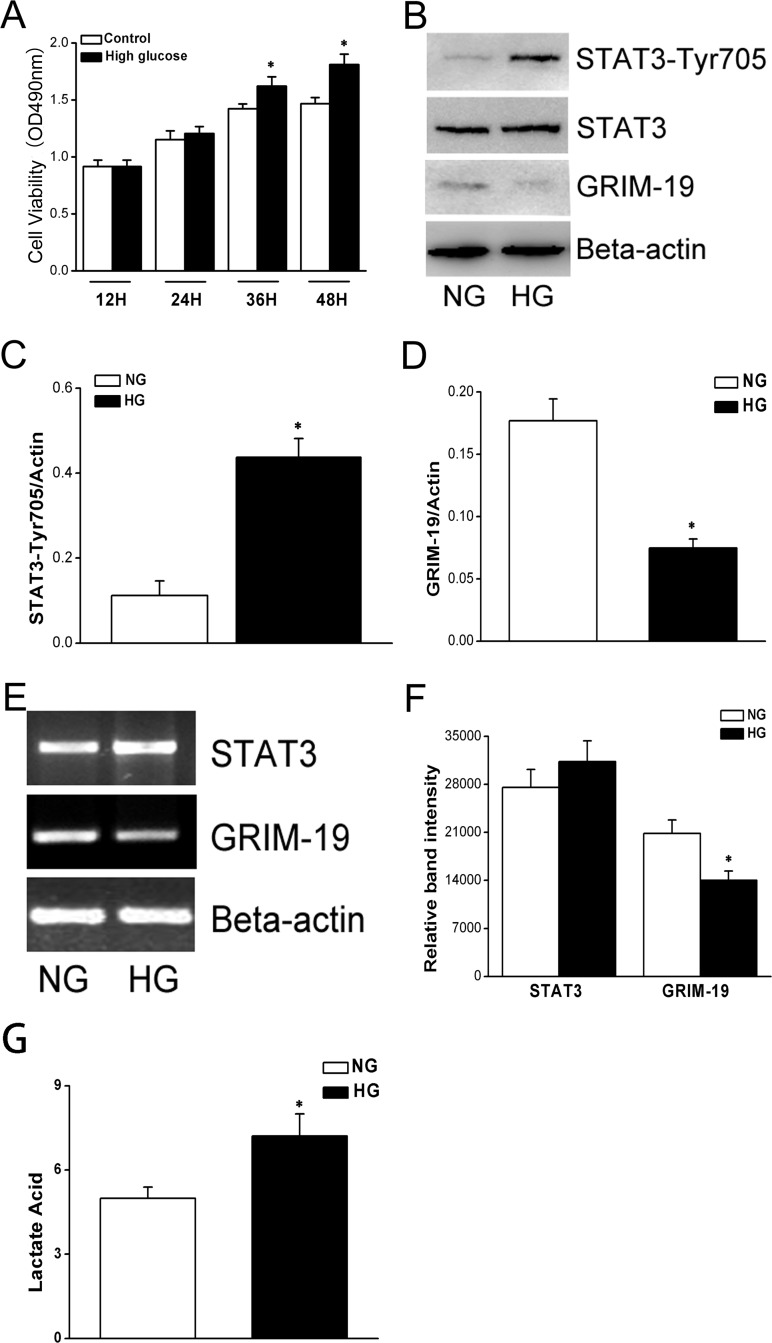
Time course changes in cell proliferation of HeLa cels in HG culture by MTT assay (shown in panel A). Both p-STAT3 over total STAT3 and GRIM-19 expression level were measured by western blotting (shown in panel B) and quantified (shown in panel C&D) where β-actin serves as a loading control. RT-PCR was performed, demonstrating that the changes in GRIM-19 and STAT3 at mRNA levels, with bands shown in panel E, and quantified in panel F. Lactate acid production was also measured in HeLa cells (G). All the data are expressed as mean±SD (three independent experiments). *, denotes P < 0.05, compared with NG group.

MTT test demonstrated that HG treatment significantly increased proliferation of HeLa cells (Fig 1A). HG treated HeLa cells had down-regulated GRIM-19 levels compared with those cultured with normal glucose medium, and this was associated with a significantly increase in STAT3 phosphorylation level at Tyrosine 705 (Fig 1B–1D). Being consistent with these, RT-PCR also showed a decrease in mRNA expression level of GRIM-19 in HG treated cells, but not that of STAT3 (Fig 1E and 1F). Interestingly, HG resulted in a significant increase in lactate acid level in HeLa cells (Fig 1G). Thus, we provided evidence that HG treated HeLA cells had enhanced proliferation, down-regulated expression of mitochondrial OXPHOS protein GRIM-19 and increased STAT3 phosphorylation level.

### GRIM-19 knock-down alone can activate STAT3 signaling and enhance HeLa cell proliferation

**Fig 2:**
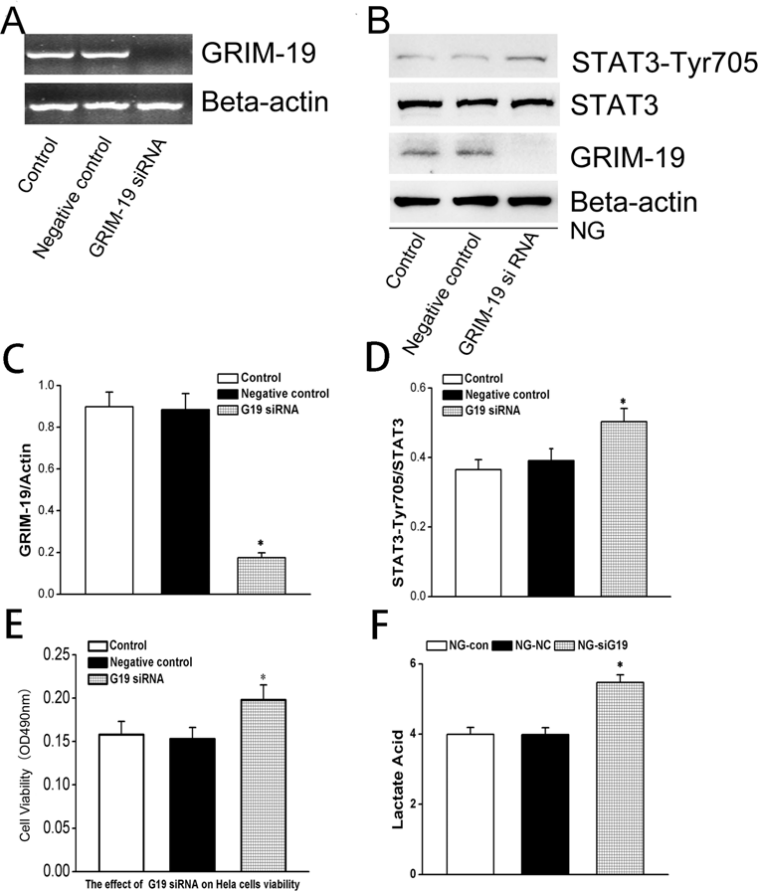
In normal glucose cultured HeLa cells, GRIM-19 was silenced by using siRNA while a scramble served as a negative control. The effects of siRNA were confirmed by RT-PCR with ß-actin serving as a control (panel A). The expression of p-STAT3/total STAT3 and GRIM-19 levels were measured by western blotting (shown in panel B) and quantified in the bar graph for GRIM-19 (C) and p-STAT3 (D) where ß-actin served as a loading control. Again, MTT assay (E) and lactate acid production (F) were performed to demonstrate the effects of knock-down of GRIM-19. Data were obtained from three independent experiments and expressed as mean±SD. *, denotes a *P*< 0.05 in comparison with the control group.

To elucidate whether down-regulated GRIM-19 was responsible for the activation of STAT3 (phosphorylation level) and hence the proliferation activity, we transfected the HeLa cells with siRNA targeting GRIM-19 to down-regulate the GRIM-19 expression. The efficiency of transfection was confirmed by the mRNA level of Grim-19 using PCR (Fig 2A). Interestingly, down-regulated GRIM-19 expression alone can increase the phosphorylation level of STAT3 when the HeLa cells were cultured in the normal glucose medium (Fig 2B–2D), indicating a causative relationship between decreased level of GRIM-19 and activation of STAT3 signaling. And the down-regulated GRIM-19 also resulted in an increase in HeLa cells proliferation as measured by MTT assay (Fig 2E). In addition, down-regulated GRIM-19 also increased lactate acid levels of HeLa cells even cultured in normal glucose medium (Fig 2F).

### Over-expression of GRIM-19 attenuated the STAT3 signaling activation induced by HG in HeLa cells

**Fig 3:**
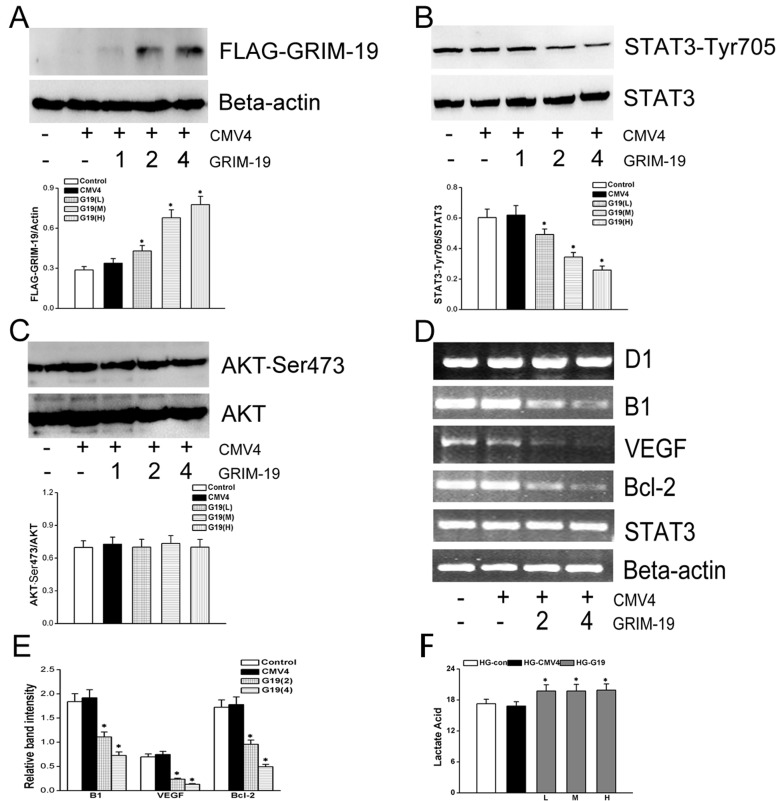
In HG cultured HeLa cells, HeLa cells were infected by lentivirus that contained a FLAG plasmid to over-express GRIM-19. The dose-dependent effects of virus infection on GRIM-19 expression were shown by measuring FLAG expression using western blotting as shown in bar graph (in panel A, ß-actin served as a loading control). Levels of p-STAT3/STAT3 (panel B), p-AKT/AKT(panel C) were measured by western blotting for HG cultured HeLa cells that over-express GRIM-19 in comparison with vector. The target genes of STAT3D, including cyclinD1, cyclinB1,VEGF and Bcl-2, were quantified by RT-PCR, with representative ethidum bromide stained gels showing in panel D and analyzed in panel E. The lactate acid production was also measured with dose-dependent effects of GRIM-19 over-expression (F). Data are expressed as mean±SD (three independent experiments). *, denotes a *P*< 0.05 compared with the control group.

We then went further to test whether over-expression of GRIM-19 can affect activated STAT3 signal induced by HG. GRIM-19 was infected into HeLa cells by lentivirus to over-express GRIM-19, showing dose-dependent increases in GRIM-19 expression (Fig 3A) which in turn attenuated increased STAT3 phosphorylation level induced by HG (Fig 3B). However, GRIM-19 over-expression alone did not affect the phosphorylation level of AKT (Fig 3C). The reversal of STAT3 signaling activation induced by HG was also evidenced by the decreases in the mRNA levels of STAT3 target genes, such as Cyclin B1, VEGF and Bcl-2 (Fig 3D and 3E), except for CyclinD1. Of note, over-expression of GRIM-19 was not associated with any changes in total STAT3 expression level. Ironically, over-expression of GRIM-19 did not reverse the increased lactate acid level induced by HG, instead, it actually increased the lactate level further compared with HG culture alone (Fig 3F).

### Grim-19 was involved in IL-6/STAT3 signaling in HeLa cells

**Fig 4:**
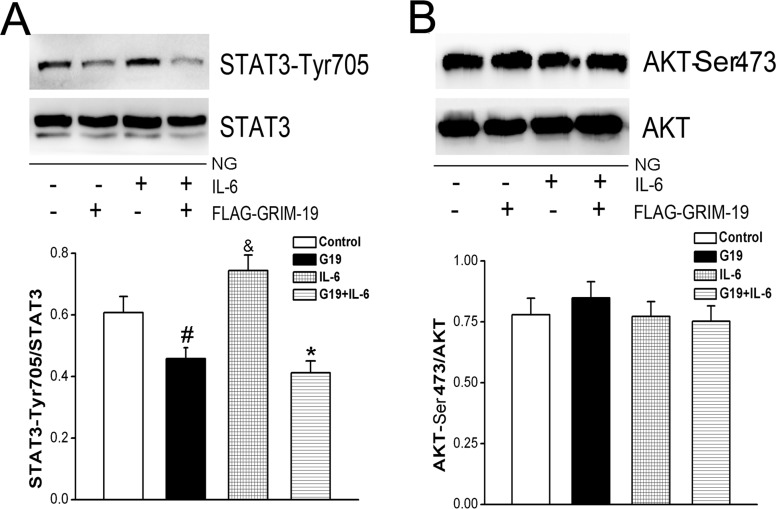
The effects of IL-6 on STAT3 activation were investigated in normal glucose cultured HeLa cells that infected with or without lentivirus to over-express GRIM-19. Both phosphorylation levels of STAT3 (shown in panel A) and Akt (shown in panel B) were measured by western blotting relative to the total expression of STAT3 or Akt, respectively. Data are expressed as the mean±SD (of three independent experiments). *, denotes a *P* < 0.05 compared with the same IL-6 stimulated HeLa cells without GRIM-19 over-expression. #, *P* < 0.05 compared with HeLa cells without GRIM-19 over-expression.&, *P* < 0.05 compared with HeLa cells without GRIM-19 over-expression or IL-6 stimulation.

We then tested whether over-expression of GRIM-19 also can suppress IL-6 activated STAT3 signaling, HeLa cells were cultured in normal glucose and exposed to IL-6 stimulation. Even though IL-6 alone did not result in any changes in Grim-19 expressions (data not shown), it significantly increased STAT3 phosphorylation levels (lane 3 vs.1, Fig 4A) with no changes in the total STAT3 expression levels. And over-expression of Grim-19 significantly decreased phosphorylation level of STAT3 in the presence (lane 4 vs. 3) or the absence of IL-6 (lane 2 vs. 1. Fig 4A). However, this did not result in any changes in AKT phosphorylation levels (Fig 4B).

### STAT3 is responsible for enhancing HeLa cells proliferation in the setting of HG culture

**Fig 5:**
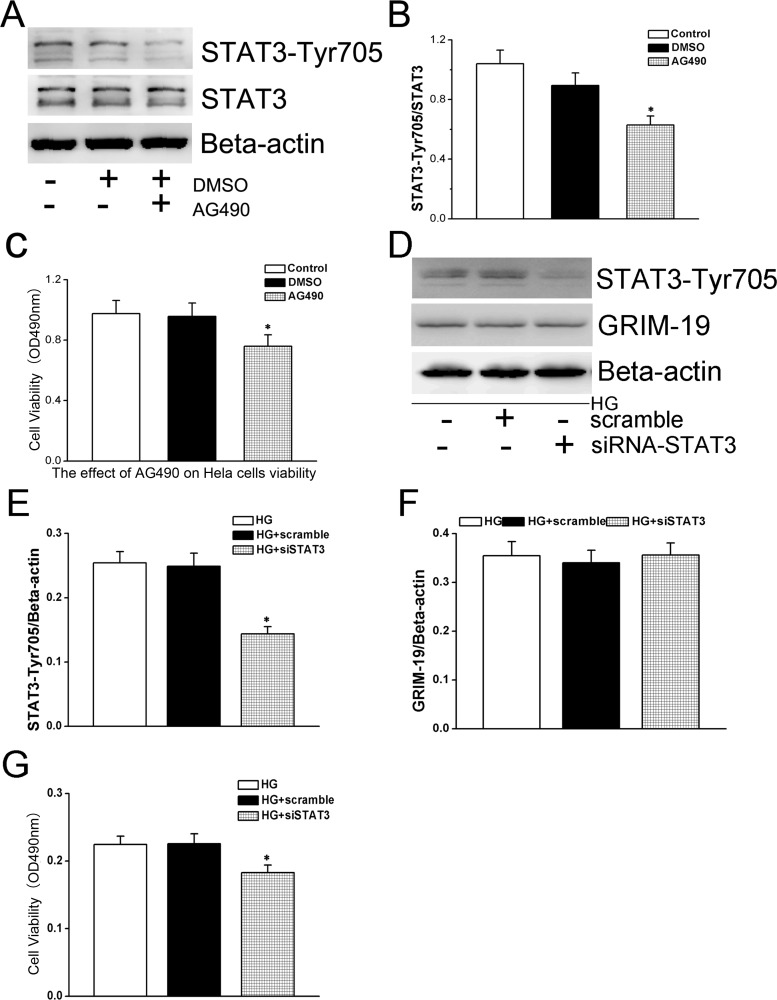
The effects of HG on STAT3 activation were tested when AG490 (40 μM) was added to the medium (DMSO as a vehicle served as a control). The STAT3-Tyr705 phosphorylation level relative to total STAT3 expression was measured by western blotting (in panel A) where ß-actin served as a loading control, and quantified in bar graph (panel B). Cell proliferation was also measured by the MTT assay (in panel C). HG cultured HeLa cells were also treated with siRNA specially targeting STAT3 with a scramble as a control. The effects of siRNA-STAT3 on phosphorylation of STAT3 were shown in panel D&E and GRIM-19 expression was also quantified in comparison with HG cultured HeLa cells without any interventions (D&F). MTT assay was performed to test the cell proliferation (G). All the data are expressed as mean±SD (of three independent experiments). * denotes *P* < 0.05 compared with group either without AG490 or siRNA targeting STAT3.

We have demonstrated down-regulated Grim-19 enhanced whereas up-regulated Grim-19 inhibited STAT3 phosphorylation levels in HeLa cells. When AG490, a JAK2 specific inhibitor, was added to the HG culture medium, it had no effects on GRIM-19 expression (data not shown), however, significantly down-regulated HG activated STAT3 phosphorylation levels (Fig 5A and 5B). Importantly, AG-490 also blocked HG induced cell proliferation (Fig 5C). To further validate the role of STAT3, we showed that knock-down of STAT3 in HeLa cells by specific siRNA (Fig 5D and 5E) can significantly decrease cell proliferation caused by HG (Fig 5G), without affecting GRIM-19 expression levels (Fig 5D and 5F).

### Activation of GRIM-19/STAT3 signaling induced by HG also occurred in H9C2 cells

**Fig 6:**
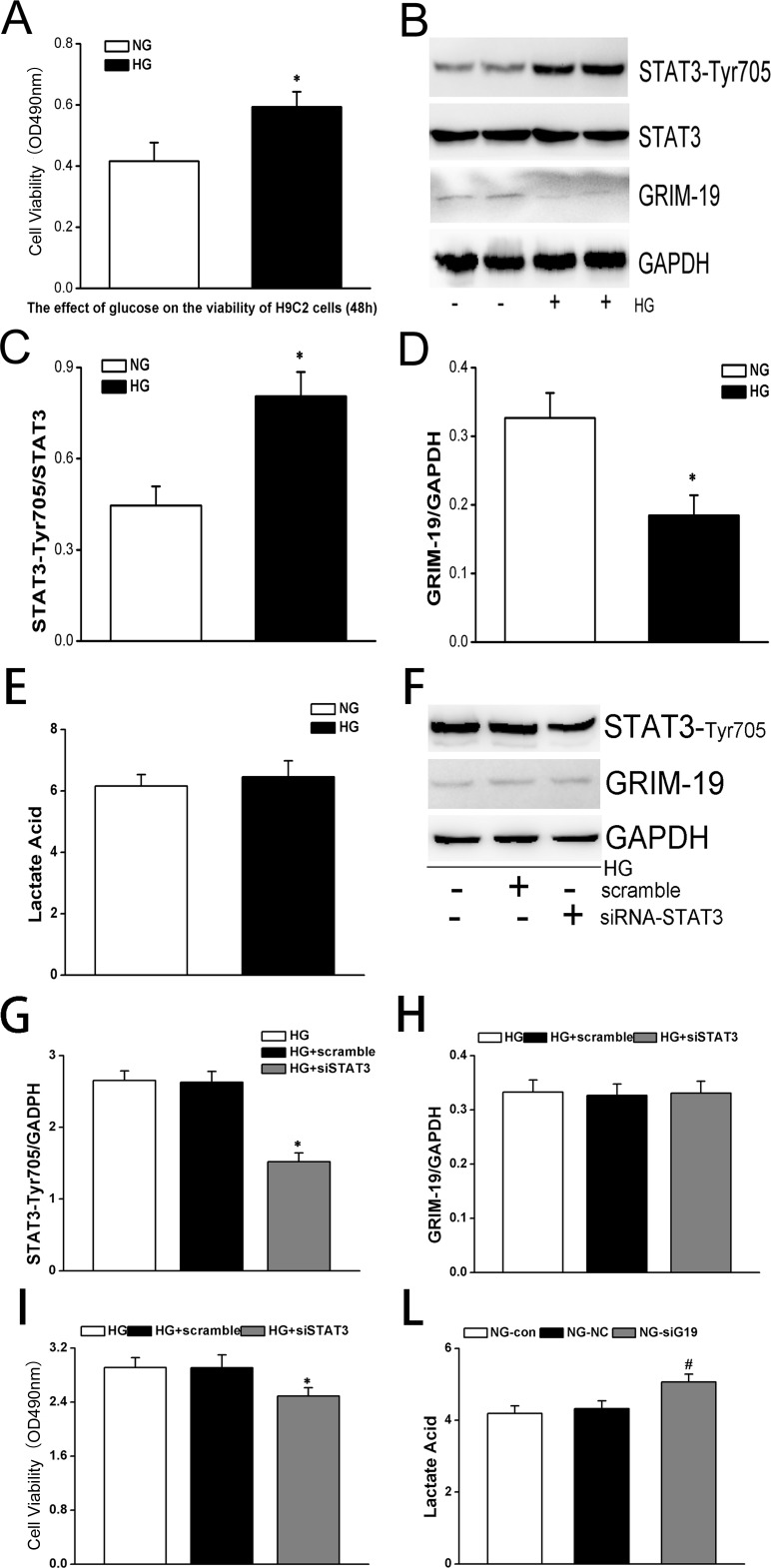
A. The effects of HG on cell proliferation was also tested in H9C2 cells compared with normal glucose (NG) cultured H9C2 cells. The change in proliferation rate was analyzed by MTT assay (A). Both p-STAT3/t-STAT3 and GRIM-19 were measured by western blotting (panel B) and analyzed in bar graph (C for STAT3 activation and D for GRIM-19). GAPDH served as a loading control. Data are expressed as mean±SD (three independent experiments). Lactate acid was measured for HG cultured H9C2 cells compared with those cultured in NG medium (E). HG cultured H9C2 cells were treated with siRNA to knock-down STAT3 with scramble serving as a negative control. The effects of knock-down of STAT3 (confirmed by western blotting to measure p-STAT3^Tyr705^, in panel F&G) on GRIM-19 expression (F&H), cell proliferation measured by MTT assay (I) and lactate acid levels (L). * denotes a *P*< 0.05 compared with the cells without any intervention as a control group.

To test how HG affects GRIM-19 expression in cardiac cells, we cultured H9C2 cardiac myoblast cells in HG medium. MTT test showed that HG treatment significantly increased H9C2 cell proliferation (Fig 6A), which was also associated with down-regulated expression level of GRIM-19 and enhanced STAT3 phosphorylation (Fig 6B and 6D), indicating that HG also activated GRIM19/STAT3 signal pathway to enhance H9C2 proliferation. Interestingly, however, HG cultured H9C2 did not show an increase in lactate acid production (Fig 6E). Knock-down of STAT3 by transfecting H9C2 cells with siRNA targeting STAT3 (Fig 6F and 6G) did not significantly affect the GRIM-19 expression level (Fig 6F and 6H), however, abolished the increased proliferation induced by HG (Fig 6I). Silence of STAT3 in H9C2 resulted in an increase in lactate acid production (Fig 6L).

**Fig 7:**
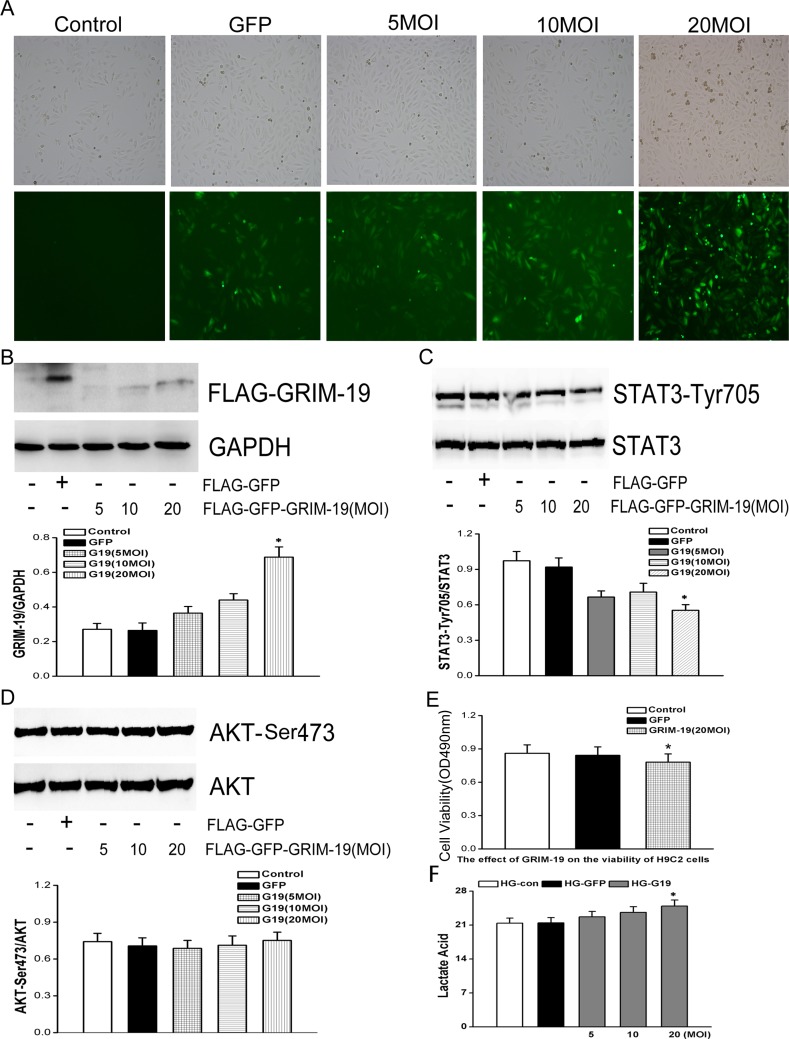
H9C2 cells were infected with lentivirus that contain both GFP and FLAG to over-express GRIM-19 (efficiency of infection was demonstrated in panel A&B). H9C2 cells were then cultured in HG medium. Then western blot (C) was done to show that the expression level of GRIM-19 dose-dependently suppressed STAT3 activation that was induced by HG (the first line of panel C). The expression level of p-AKT/total Akt was not affected by GRIM-19 (panel D). MTT assay (E) and lactate acid level (F) were investigated to evaluate the dose-dependent effects of GRIM-19 over-expression. Data are obtained from three independent experiments and expressed as mean±SD. * denotes a *P < 0*.*05*, compared with the H9C2 with HG culture medium.

In contrast, over-expression of GRIM-19 in H9C2 cells by lentiviral infection (Fig 7A and 7B) can also attenuate increased STAT3 phosphorylation levels induced by HG (Fig 7C), without affecting AKT phosphorylation levels (Fig 7D). This also resulted in a significant decrease in H9C2 cells proliferation in the setting of HG (Fig 7E), further suggesting that activation of GRIM-19/STAT3 signaling facilitated H9C2 proliferation. Over-expression of GRIM-19 dose-dependently increased lactate acid levels in H9C2 (Fig 7F).

### Roles of AMP- activated kinase in HG induced changes in GRIM-19 expression in HeLa and H9C2 cells

**Fig 8:**
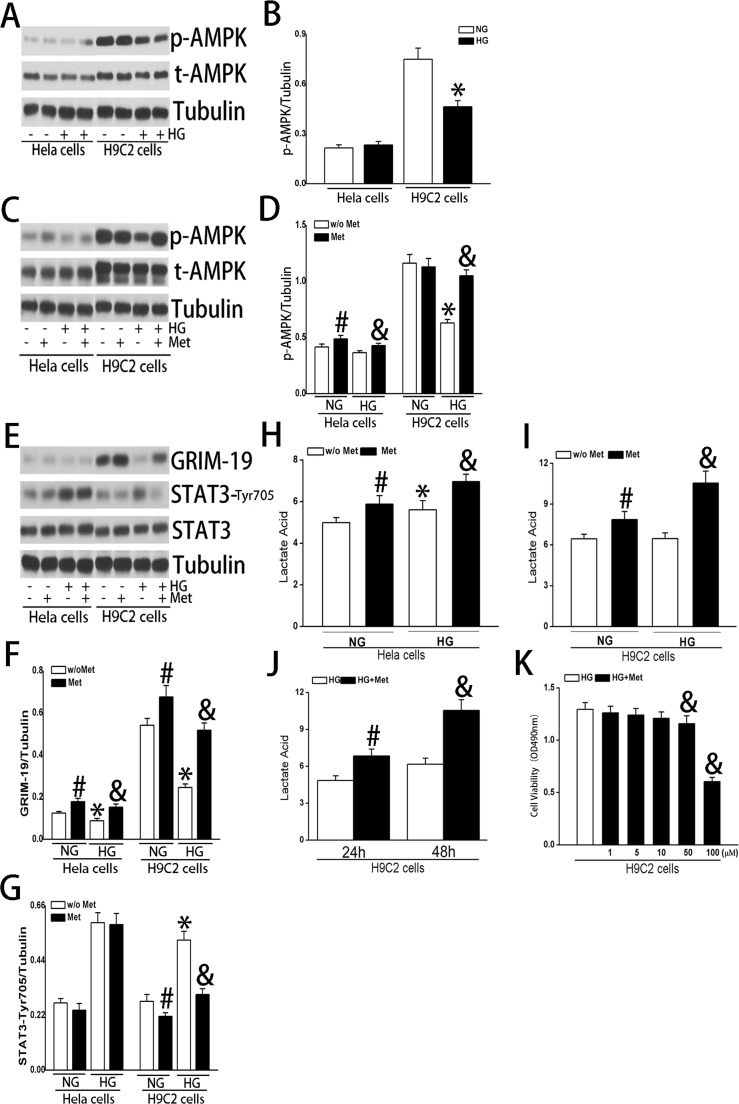
The effects of HG on AMPK phosphorylation were tested in both HeLa and H9C2 cells (A and B) and the effects of metformin on phosporylation of AMPK in the setting of HG were also tested (C and D). The effects of metformin on phosphorylation level of STAT3 and GRIM-19 were also quantified for both HeLa and H9C2 cells (E-G). The effects of metformin on lactate acid production were also evaluated for both HeLa cells (H) and H9C2 (I) cells. The time dependent (J) and dose dependent effects of metformin on lactate acid production were also demonstrated in H9C2 cells. Data are obtained from three independent experiments and expressed as mean±SD. * denotes a *P < 0*.*05*, compared with cells with NG culture medium; # *P < 0*.*05* compared with HG cultured cells without metformin.

To further test whether AMPKα activity was involved in HG induced down-regulated GRIM-19 expression, we then measured AMPKα activation to evaluate its interactions with GRIM-19 in the setting of HG. Intriguingly, HG cultured HeLa cells did not exhibit any change in AMPKα phosphorylation level, however, a significant decrease in phosphorylated AMPKα level was present in HG cultured H9C2 cells (Fig 8A and 8B). Metformin administration only slightly increased phosphorylation level of AMPKα in HeLa cells in both normal glucose and HG culture condition. However, metformin resulted in a recovery of the down-regulated phosphorylted AMPKα2 level caused by HG culture in H9C2 cells, even though had no significant effects in normal glucose culture condition (Fig 8C and 8D). In addition, metformin not only slightly increased GRIM-19 expression in normal glucose cultured HeLa and H9C2 cells, but also resulted in a complete recovery of GRIM-19 expression that was suppressed by HG (Fig 8E and 8F). Interestingly, while metformin had no effects on STAT3 signaling in HeLa cells, however, it significantly abolished the increased STAT3 phosphorylation in HG cultured H9C2 cells (Fig 8E and 8G). In addition, metformin increased lactate acid in both HeLa and H9C2 cells cultured either in normal glucose or in HG (Fig 8H and 8I), showing time dependent effects in H9C2 cells (Fig 8J) which was associated with suppressed cell proliferation again in a dose-dependent fashion (Fig 8K).

## Discussion

Our study demonstrates for the first time that HG can down-regulate the mitochondrial protein GRIM-19 expression in both HeLa and H9C2 cells, which in turn can activate STAT3 signaling, leading to enhanced phosphorylation level of STAT3 and cell proliferation capability of both HeLa cells and H9C2 cells. Importantly, GRIM-19 silencing alone with normal glucose culture resulted in similar effects as HG culture. In contrast, over-expression of GRIM-19 attenuated p-STAT3 activation induced by HG and resulted in a decrease in cell proliferation for both HeLa and H9C2 cells. In addition, side by side comparison was made between HeLa and H9C2 cells and a strikingly different expression pattern of changes was shown in AMPK activation,showing a decrease in AMPKα phosphorylation levels by HG culture in H9C2 cells, but no change in HeLa cells. In contrast, HG induced lactate secretion was only observed in HeLa cells, not in H9C2 cells. Metformin only had trivial effects on modulating AMPKα activities in HeLa cells, but significantly reversed down-regulated AMPKα phosphorylation levels induced by HG in H9C2 cells. Of note, metformin also reversed down-regulated GRIM-19 expression and up-regulated STAT3 activation both of which were caused by HG culture, however, only slightly increased GRIM-19 expression, yet had no effects on STAT3 phosphorylation levels in HeLa cells.

STAT3 as an important transcription factor has long been recognized as a mediator for regulating its targeted genes in response to extracellular stimuli, thus playing key roles in various cellular biological processes such as cell growth and apoptosis. It has been well established that STAT3 can be activated in cancer cells, which makes itself an attractive target for anti-cancer therapy[22]. Interestingly, activated STAT3 signaling has been involved in insulin resistance [15]. STAT3 activation was induced in response to innate inflammation in the setting of high free fatty acid in diabetic setting [15]. In our present study, we found that high glucose can up-regulate the p-STAT3 level in both HeLa and H9C2 cells, which was also observed in HepG2 cells in our previous work [16]. In fact, it was GRIM-19 that was responsible for STAT3 activation induced by HG. The role for GRIM-19 in STAT3 activation was further confirmed by the data that down-regulated Grim-19 expression alone in normal glucose culture can mimic the effects of HG, and HG induced activation of STAT3 signaling can be blocked by GRIM-19 over-expression. Moreover, AG490 or siRNA targeting STAT3 can attenuate the enhanced cell proliferation in the setting of HG. Thus, our data strongly support the concept that Grim-19/STAT3 mediates HG induced cell proliferation for both HeLa and H9C2 cells.

GRIM-19 as one important mitochondrial OXPHOS protein has already been shown to be able to gear metabolic states through modulating STAT3/HIF1α signal [10]. Chen et al. reported that a down-regulated GRIM-19 expression can impair the mitochondrial complex I activity, leading to an increase in reactive oxygen species generation[23]. In contrast, over-expression of GRIM-19 can markedly suppress inflammatory cytokine levels such as IL-6 and tumor necrosis factor-α in arthritic joints[24]. In cancer cells, GRIM-19 expression was actually down- regulated in cancer cells[11,25], and its level was inversely correlated with phosphorylation level of STAT3, suggesting that a lower GRIM-19 level favors or promotes tumor growth via promoting STAT3 biological activity [12,26]. At present stage, we still don’t know how down-regulated Grim-19 expression can activate STAT3 signaling. We can only assume that down-regulated Grim-19 expression resulted in impaired mitochondrial complex-I function which resulted in compromised mitochondrial function and activate the function of STAT3, favoring proliferation and/or survival of cells. Thus, a link between high glucose and Grim-19/STAT3 signaling has been observed. It should be noted that other parallel pathways, such as Shp-1 as an important phosphatase, could be independently involved in regulating STAT3 activation in the setting HG. It would be interestingly to test whether a higher STAT3 activity would be also observed in patients with a high glucose level such as cancer patients with diabetes or obesity.

In fact, STAT3 activation in cancer cells is closely related to mitochondrial pathway [27]. It has previously been shown that the mitochondrial uncoupling protein 2 regulates the ROS/Stat3 signaling pathway and responds to the chemotherapy in lung cancer cells [27]. Interaction between STAT3 and Grim-19 is important for optimal function of mitochondrial complex I of electro transfer chain, which determines the appropriate amount of reactive oxygen species(ROS) to promote breast cancer growth [28]. It was proposed that mitochondria might function as a central checkpoint by integrating various signals coming either from endogenous elements (such as ions, metabolites or even second messengers) and/or signaling proteins (such as kinases and phosphatases), or from exogenous factors (nutrients, oxygen). Thus, in the setting of HG, down-regulated Grim-19 could serve as a sensor and transmit the signaling of mitochondria to the nucleus through its interaction with STAT3, promoting the survival of cancer cells or proliferation of the cells such as H9C2. It will be also interesting to test if other components of mitochondria would function the same way as Grim-19 [29].It certain warrants further studies that aim to elucidate whether this would be the same case for other types of cells.

In our study, we also evaluated interaction between AMPK and GRIM-19, specifically whether AMPKα activity, an important sensor for energy status [30], would be involved in this HG induced down-regulated GRIM-19 expression. We have demonstrated strikingly different patterns of changes in AMPK activities between HeLa and H9C2 cells. We observed a low expression level of AMPKα in the HeLa cells, whereas in H9C2 cells AMPKα expression is relatively much higher, indicating different metabolic status between these two cell lines. This could explain why HG resulted in a significant decrease in AMPKα activity in H9C2 cells in comparison to much lower fold of changes in HeLa cells, highlighting the importance of AMPKα in modulating the metabolic regulation in cardiac cell line. Interestingly, metformin can abolish the changes induced by HG culture, i.e. reversal of down-regulated GRIM-19 and activated STAT3 signaling that were seen in HG cultured H9C2 cells, whereas in HeLa cells only slight increase in GRIM-19 was observed, without any changes in STAT3 phosphorylation status. Thus, our data not only confirm previous study that AMPK could regulate the activity of STAT3 [31] in cardiac cells, but also elucidate that it was GRIM-19 which mediates activation of STAT3 signaling.

### Study limitations

There are several limitation in this study we have to discuss. First, it has been well established that lactate functions as an important metabolic player in cancer cells [32]. In our study, we observed HG culture elevated lactate secretion in HeLa cells compared with normal glucose culture, however, not H9C2 cells. Thus, lactate secretion induced by HG was closely correlated with enhanced cell proliferation, which was associated with down-regulated GRIM-19 expression. Meanwhile, it is difficult to interpret that up-regulated GRIM-19, on the other hand, also increased lactate secretion. Thus, relationship between lactate secretion and GRIM-19 expression cannot be clearly defined in our present study. Second, using metformin as a pharmacological tool, we also looked at the potential roles for AMPK in GRIM-19/STAT3 signaling pathway in the setting of HG culture, even though we observed a strikingly different pattern of changes in AMPK activity in HeLa and H9C2 cell lines in response to HG culture and metformin intervention (Fig 8H and 8I), we need to be aware of that non-specific effects of metformin cannot be excluded. A genetic approach is certainly needed to further elucidate the role of LKB1/AMPK pathway. Third, our data indicated that AKT and STAT3 showed differential response to IL-6 stimulation, as AKT was not activated when cells were exposed to IL-6 stimulation which was strikingly different from the previous data. We reasoned that it could be due to the normal glucose cell culture was selected as a control, whereas the cells are usually cultured in HG medium in most of the experimental studies where the effects of IL-6 were investigated. Fourth, it remains unknown why metformin up-regulated GRIM-19 expression in both HeLa and H9C2 cells, which resulted in a normalization of STAT3 activation in H9C2 cells, however, failed to decrease STAT3 phosphorylation levels in HeLa cells. We reason that it could be due to different levels of AMPK activation and their responses to metformin therapy in these two cells levels. If this holds true, i.e., AMPK plays a key role in differentiating these two types of cells in responses to metformin, then it will be interesting to test whether knock-out or depletion of GRIM-19 affects metformin-mediated inhibition of p-STAT3 in H9C2 cells. Finally, it remain unknown how activated AMPK by metformin increase GRIM-19 expression in H9C2. Moreover, it is still not understood why AMPK level is relatively lower in HeLa cells than in H9C2. In fact the role of AMPK in cancer cells is still highly controversial [33]. Further studies are certainly warranted to answer these important questions.

In conclusion, the mitochondrial complex I protein GRIM-19 can mediate hyperglycemia-induced p-STAT3 signal in both HeLa and H9C2 cell culture. In H9C2 cells, GRIM-19 is involved in metformin enhanced AMPKα activation that is suppressed by HG, which in turn down-regulates STAT3 activity that is activated by HG. Further study designed to look further into GRIM-19 mediated mitochondrial pathway may cast new lights on the treatment of various HG-associated diseases, such as diabetes and tumors.

## Abbreviations

GRIM-19: Gene associated with retinoid-IFN-induced mortality 19
HG: High Glucose
STAT3: Signal transducer and activator of transcription 3
MTT: 3-(4, 5-dimetrylthiazol-2-yl)-2, 5-diphenyltetrazolium bromide
AMPK: AMP-activated protein kinase
